# Mitochondrial DNA and karyotypic data confirm the presence of *Mus indutus* and *Mus minutoides* (Mammalia, Rodentia, Muridae, *Nannomys*) in Botswana

**DOI:** 10.3897/zookeys.359.6247

**Published:** 2013-12-05

**Authors:** Molly M. McDonough, Cibele G. Sotero-Caio, Adam W. Ferguson, Patrick J. Lewis, Matlhogonolo Tswiio, Monte L. Thies

**Affiliations:** 1 Department of Biological Sciences, Texas Tech University, Lubbock, Texas, 79409-3131, USA; 2 Department of Biological Sciences, Sam Houston State University, Huntsville, Texas, 77341, USA; 3 Department of National Museums and Monuments, Gaborone, Botswana

**Keywords:** Africa, rodent, distribution, karyotype, sex-autosome translocation, cytochrome *b*

## Abstract

We use a combination of cytochrome *b* sequence data and karyological evidence to confirm the presence of *Mus indutus* and *Mus minutoides* in Botswana. Our data include sampling from five localities from across the country, including one site in northwestern Botswana where both species were captured in syntopy. Additionally, we find evidence for two mitochondrial lineages of *M. minutoides* in northwestern Botswana that differ by 5% in sequence variation. Also, we report that *M. minutoides* in Botswana have the 2n=34 karyotype with the presence of a (X.1) sex-autosome translocation.

## Introduction

Delineating geographic distributions of African *Mus* (subgenus *Nannomys* Peters, 1876) in Sub-Saharan Africa has been especially challenging due to a combination of incomplete taxon sampling throughout the region as well as uncertainties in species identification resulting from their highly conserved morphology. Despite morphological similarities, African pygmy mice (*Nannomys*) are characterized by a high degree of chromosomal variation, including chromosomal rearrangements such as Robertsonian translocations, pericentric inversions, heterochromatin additions, and tandem fusions (see summary in Britton-Davidian et al. 2012). Additionally, whole-arm translocations (WARTs) and novel sex-chromosome determination have been documented in populations in South Africa (Veyrunes et al. 2007, 2013).

Britton-Davidian et al. (2012) produced the most complete phylogenetic analysis of *Nannomys* to date, which included previously published sequences of nine species (Suzuki et al. 2004; Chevret et al. 2005; Veyrunes et al. 2005; Kan Kouassi et al. 2008; Veyrunes et al. 2010; Mboumba et al. 2011; and others). Their phylogeny illuminated the diversity of taxa within this subgenus (including at least one unnamed species from Chad), and clearly indicated that further phylogenetic investigations are necessary to clarify species diversity within *Nannomys*. Their comprehensive review surmised that there are at least 18 species of African pygmy mice and estimated that eight species occur within southern Africa (Britton-Davidian et al. 2012). In addition, theirstudy highlighted important gaps in both geographic and taxonomic sampling for this subgenus, particularly within southern Africa. Included in this underrepresented southern African group is *Mus minutoides*, one of the most widespread pygmy mice, with a distribution encompassing most of Sub-Saharan Africa.

**Figure 1.**
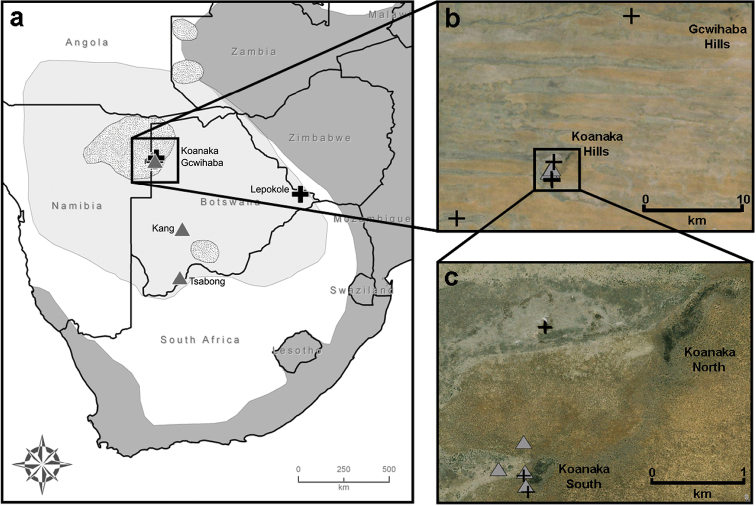
Distributions for three species of *Nannomys* in southern Africa. Dark grey indicates distribution for *Mus minutoides*, light grey for *Mus indutus*, and stippled pattern for *Mus setzeri*, adapted from Monadjem (2008a), Monadjem (2008b), and Monadjem and Coetzee (2008), respectively. Five trapping localities in Botswana (**a**); black crosses indicate captures for *Mus minutoides* and grey triangles for *Mus indutus*. Records from northwestern Botswana, Ngamiland District (**b**). Locality of syntopic records for *Mus indutus* and *Mus minutoides* at Koanaka Hills site (**c**).

Within the southern African country of Botswana, the taxonomy of *Mus* has never fully been resolved. Early assessments of the regional mammalian fauna (Smithers 1971) concluded that two native forms of *Mus* exist within Botswana: the widespread *Mus minutoides indutus* (Thomas, 1910)—which was later elevated to specific status (Petter and Matthey 1975; Musser and Carleton 1993, 2005)—and an arid-adapted form with large ears and a white band of fur near the rump (referred to as *Leggada* sp. in Smithers 1971) restricted to northwestern Botswana and a single record from Sekhuma Pan in the Jwaneng District of southern Botswana (Petter 1978). This latter species was later described as *Mus setzeri* Petter, 1978. De Graaff’s (1981) assessment of *Nannomys* in southern Africa concluded that all records for Botswana conform to *Mus minutoides*, although he acknowledged that *Mus minutoides indutus* and the desert form (*Mus setzeri*) may be distinct species that require further study. More recent evaluations describe allopatric distributions for *Mus indutus* and *Mus minutoides* and only acknowledge the former within the boundaries of Botswana (Skinner and Chimimba 2005, Happold and Veyrunes 2013). These recent assessments estimated the geographic range for *Mus minutoides* as extending from the southwest cape in South Africa through the Zambezian woodlands in the east (Fig. 1a, dark grey). Monadjem (2013a) stated that *Mus indutus* replaces *Mus minutoides* in the western part of the Zambezian woodlands and extends throughout Botswana and into neighboring countries (Fig. 1a, light grey). Although Britton-Davidian et al. (2012) proposed that the range of *Mus minutoides* greatly differs from the map published by Monadjem (2008b), and including the countries of Angola, Botswana, Namibia, Zambia, and Zimbabwe, verified records from their study were only presented for South Africa, Swaziland, and Zimbabwe. However, records of *Mus minutoides* have recently been confirmed for Angola and Namibia (Lamb et al. 2014), providing additional support for the extended range map proposed by Britton-Davidian et al. (2012)

Regarding chromosomal rearrangement in southern Africa, *Mus minutoides* from South Africa exhibit Robertsonian fusions with two major monophyletic groups showing either a diploid number of 2n=18 – where all of the acrocentric chromosomes are fused to produce metacentric elements, or a 2n=34 – where sex-chromosome translocations have been reported (Veyrunes et al. 2010). Additionally, WARTs have been documented in several populations exhibiting the 2n=18 karyotype in South Africa, which has contributed significantly to reported chromosomal variation, with at least four different cytotypes within this clade (Veyrunes et al. 2007). Currently, the geographic distributions of the 2n=18 and 2n=34 forms of *Mus minutoides* are not known outside of the country of South Africa (Veyrunes et al. 2010).

Our objective was to utilize material from recent collecting efforts and molecular techniques to accurately delimit which species of *Nannomys* occur within Botswana. Further, we describe karyotypes for individuals from this region and make comparisons with previously published data from South Africa.

## Materials and methods

Our mitochondrial phylogeny was generated from combining previously published sequences deposited on GenBank (Appendix) with those derived from sequencing new specimens collected during field trips to Botswana conducted in 2008, 2009, and 2011 (Table 1, Appendix). We collected 16 specimens of *Mus* from five localities in Botswana including: Gcwihaba Caves (20°00.99'S, 21°15.89'E); Kang (23°32.10'S, 22°32.76'E); Koanaka Hills (20°09.60'S, 21°11.61'E); Lepokole Hills (21°49.59'S, 28°23.94'E); and Tsabong (25°56.57'S, 22°25.44'E) (Fig. 1a–c). Specimens were collected using Sherman live traps, pitfall traps, or Museum Special snap traps. Standard external measurements were recorded in the field (Table 1). Specimens were preserved as skins with complete skeletons (SSPS), skulls only, or as whole bodies in alcohol (alc.) and deposited at the at the Natural Science Research Laboratory (NSRL) at the Museum of Texas Tech University, Lubbock, Texas, USA or the Botswana National Museum, Gaborone, Botswana. Tissue samples were preserved in 95% ethanol, lysis buffer, or flash frozen in liquid nitrogen for future genomic analyses (2011 material) and deposited in the NSRL. Field collecting methods followed taxon specific guidelines for wild mammals (Sikes et al. 2012) as outlined by the Animal Care and Use Committee of the American Society of Mammalogists (Gannon et al. 2007; Sikes et al. 2011).

**Table 1.** Locality information for 16 specimens of *Mus (Nannomys)* collected in Botswana during June 2008, July 2009, and August 2011. Verbatim coordinates were recorded in the field using a handheld Garmin GPS Rino 120 unit using the datum WGS84. Elevations given in meters.

Tissue No.	Genbank No.	Species	District	Specific Locality	Verbatim Coordinates	Verbatim Coordinate System	Verbatim SRS	Verbatim Elevation	Latitude, Longitude	Elev.	Coordinate Uncertainty
TK170604	KF184321	*Mus indutus*	Kgalagadi	Berry Bush Farm, 8 km N, 2 km E Tsabong (Tshabong)	-25.94283, 22.42405	Decimal degrees	WGS84	971	25°56.57'S, 22°25.44'E	970	31.5 m
TK172845	KF184320	*Mus indutus*	Kgalagadi	Berry Bush Farms, 8 km N, 2 km E Tsabong (Tshabong)	-25.94283, 22.42405	Decimal degrees	WGS84	971	25°56.57'S, 22°25.44'E	970	31.5 m
TK172826	KF184322	*Mus indutus*	Kgalagadi	Kalahari Rest, 16 km N, 25 km W Kang	-23.53498, 22.54607	Decimal degrees	WGS84	1158	23°32.10'S, 22°32.76'E	1160	31.5 m
TK172785	KF184310	*Mus minutoides*	Central	Lepokole Hills, 3.6 km S, 4.9 km E Lepokole Village	-21.82653, 28.39898	Decimal degrees	WGS84	784	21°49.59'S, 28°23.94'E	780	31.5 m
TK164851	KF184309	*Mus minutoides*	Ngamiland	Koanaka Hills (Ncqumtsa Hills), 150 km W Tsao (Tsau), water hole	34K 0511309 7767149	UTM	WGS84	1019	20°11.58'S, 21°06.49'E	1020	31.5 m
TK154612	KF184311	*Mus minutoides*	Ngamiland	Koanaka Hills (Ncqumtsa Hills), 150 km W Tsao (Tsau), main camp	34K 0520241 7770802	UTM	WGS84	1024	20°09.60'S, 21°11.62'E	1020	31.5 m
TK164817	KF184316	*Mus indutus*	Ngamiland	**"**	34K 0520219 7770803	UTM	WGS84	1021	20°09.60'S, 21°11.61'E	1020	31.5 m
TK164820	KF184318	*Mus indutus*	Ngamiland	**"**	34K 0520219 7770803	UTM	WGS84	1021	20°09.60'S, 21°11.61'E	1020	31.5 m
TK164753	KF184319	*Mus indutus*	Ngamiland	**"**	34K 0520210 7770958	UTM	WGS84	1020	20°09.51'S, 21°11.60'E	1020	31.5 m
TK164752	KF184312	*Mus minutoides*	Ngamiland	**"**	34K 0520198 7770976	UTM	WGS84	1020	20°09.51'S, 21°11.60'E	1020	31.5 m
TK164751	KF184315	*Mus indutus*	Ngamiland	**"**	34K 0519948 7770988	UTM	WGS84	1022	20°09.50'S, 21°11.45'E	1020	31.5 m
TK164757	KF184323	*Mus indutus*	Ngamiland	**"**	34K 0519948 7770988	UTM	WGS84	1022	20°09.50'S, 21°11.45'E	1020	31.5 m
TK164939	KF184317	*Mus indutus*	Ngamiland	**"**	34K 0520201 7771287	UTM	WGS84	1027	20°09.34'S, 21°11.60'E	1030	31.5 m
TK164768	KF184313	*Mus minutoides*	Ngamiland	**"**	34K 0520416 7772600	UTM	WGS84	1026	20°08.62'S, 21°11.72'E	1030	31.5 m
TK164769	KF184308	*Mus minutoides*	Ngamiland	**"**	34K 0520408 7772612	UTM	WGS84	1020	20°08.62'S, 21°11.72'E	1020	31.5 m
TK164967	KF184314	*Mus minutoides*	Ngamiland	Gcwihaba Caves, 18.8 km N, 114.2 km W Tsao (Tsau)	34K 0527701 7786660	UTM	WGS84	986	20°00.99'S, 21°15.89'E	990	31.5 m

Genomic DNA was extracted using a DNeasy Blood and Tissue Kit (Qiagen Inc., Chatsworth, California). The complete cytochrome *b* gene (*cytb*, 1140 nucleotides) was amplified following methods outlined in Veyrunes et al. (2010). Cycle sequencing reactions were performed with BigDye terminator version 3.1 and were electrophoresed on an ABI 3100-*Avant* (Applied Biosystems, Foster City, California). Sequences were edited and aligned using SEQUENCHER version 4.9 (Gene Codes Corporation, Ann Arbor, Michigan). Novel sequences (GenBank accession nos. KF184308-KF184323) were aligned with previously published sequences deposited on GenBank using only individuals that exhibited unique haplotypes (Appendix). The final alignment was trimmed to exclude regions with large amounts of missing data due to the large number of GenBank sequences in the alignment that were partial *cytb* sequences. Therefore, a total of 741 base pairs of the *cytb* gene (the first 7 codons and last 126 codons were removed from the analysis) were used in the final alignment for the phylogenetic analysis including 125 individuals.

Appropriate models of evolution were examined using MEGA version 5 (Tamura et al. 2011). Phylogenetic relationships were estimated using Bayesian inference with the program MRBAYES version 3.2 (Huelsenbeck and Ronquist 2001). Four independent Markov chains were run for 50 million generations and trees were logged every 1000^th^ iteration. Log-likelihood values were examined in the program TRACER version 1.5 (Rambaut and Drummond 2007) and the first 5,000 trees were discarded as burn-in. An additional phylogeny was estimated using the Maximum-likelihood method with the program PhyML version 3.0 (Guindon et al. 2010) with a BIONJ starting tree (Gascuel 1997) and 1,000 bootstrap replicates. Kimura 2-parameter genetic distances were calculated using MEGA version 5 (Tamura et al. 2011).

Specimens were karyotyped in the field using bone marrow after 1 h of *in vivo* incubation with Velban (Sigma-Aldrich, St. Louis, Missouri), following the methods described in Baker et al. (2003). *Mus indutus* males were not karyotyped in this study because both males captured died in snap traps. Fluorescent *in situ* hybridization (FISH) experiments were performed using Star*FISH © biotin-labeled mouse chromosome X paints (Cambio), following the manufacturer’s instructions and using Cy3-conjugated streptavidin (Invitrogen) for signal detection.

In order to assess the nature of the X-autosome translocation of the specimens that exhibited the translocation, we compared the X-chromosome of our specimens with those from South Africa using images of inverted DAPI-banding, and G-banding (Seabright 1971). Images were captured using the GENUS SYSTEM version 3.7 (Applied Imaging Systems, San Jose, California) through an Olympus BX51 epi-fluorescence microscope. Cy3 and DAPI (4’,6-diamidino-2-phenylindole) signals were pseudocolored yellow and red, respectively.

## Results

**Figure 2.**
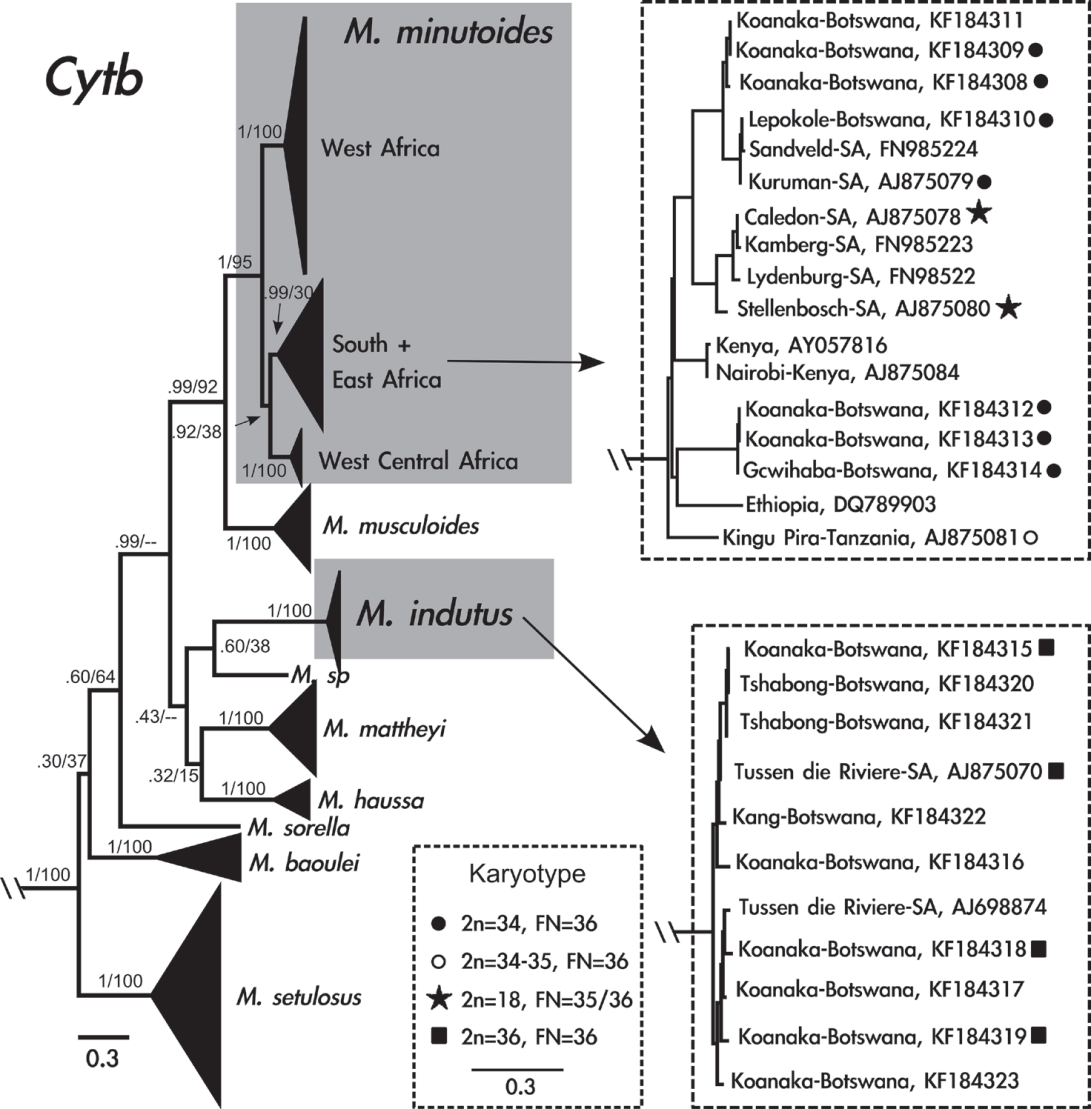
Cytochrome *b* gene tree generated from 741 base pairs including 125 taxa using Bayesian inference. Grey boxes indicate species of interest: *Mus minutoides* and *Mus indutus*. Clades that include *Mus* from Botswana are enlarged to the right of the phylogeny. Diploid and fundamental numbers are shown for individuals sampled in this study and Veyrunes et al. (2005). Identification includes GenBank number and general locality. Support values at nodes are Bayesian posterior probabilities followed by Maximum-likelihood bootstrap support; dashes indicate regions of the tree where Maximum-likelihood analysis resulted in a polytomy.

The model with the lowest AICc (Akaike Information Criterion, corrected) and BIC (Bayesian Information Criterion) scores was the General Time Reversible (GTR) model using a discrete gamma distribution (+G) and a fraction of invariable sites (+I). Overall, the two methods of phylogenetic analysis resulted in similar tree topologies, except that the Maximum-likelihood analysis recovered weak support for the south + east *Mus minutoides* clade (Fig. 2). Additionally, the relationship between *Mus indutus*, *Mus* sp., *Mus mattheyi*, *Mus haussa*, and the portion of the phylogeny that includes *Mus minutoides* and *Mus musculoides* was unresolved in the Maximum-likelihood analysis, though it was well-supported using Bayesian inference.

Sixteen *cytb* sequences were generated from specimens from Botswana, corresponding to two species. Seven individuals are phylogenetically related to *Mus minutoides* from South Africa and nine individuals cluster with *Mus indutus*. Five individuals, captured from the same locality in the Koanaka Hills region of northwestern Botswana, represent two clades within *Mus minutoides* that are 5% different in *cytb* sequence variation (Fig. 2). Six of the individuals of *Mus indutus* were collected in the Koanaka Hills alongside both of these lineages of *Mus minutoides* (Fig. 1c).

**Figure 3.**
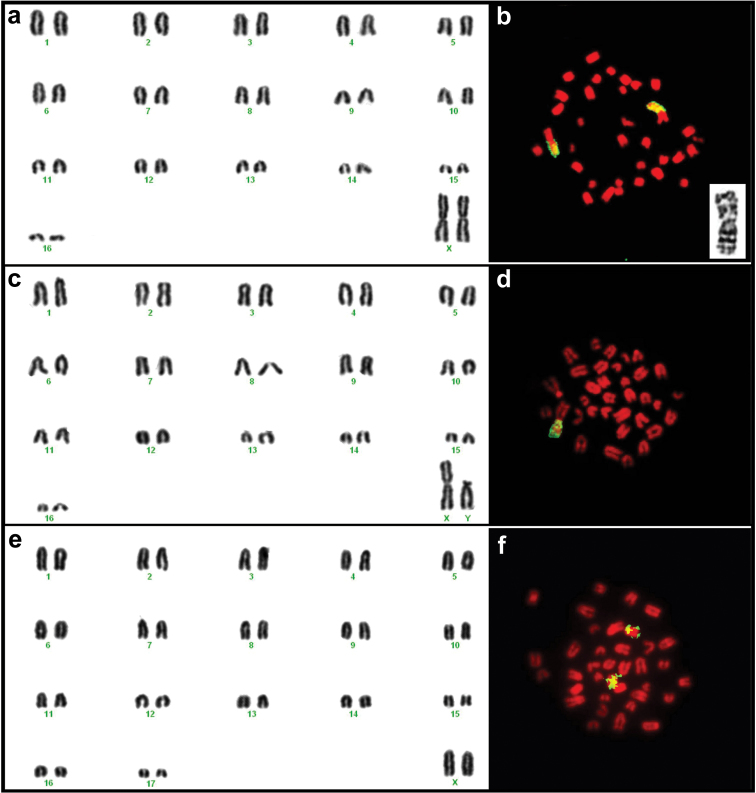
Karyotypes of female TK164752 (**a**) and male TK164768 (**c**) *Mus minutoides* and female TK164753 *Mus indutus* (**e**) from Botswana. The chromosome arms identified in yellow on the images to the right of each karyogram correspond to regions of homology to the X chromosome of *Mus musculus* detected by FISH for female TK164752 (**b**) and male TK164768 (**d**) *Mus minutoides* and female TK164820 *Mus indutus* (**f**). Note that in *Mus minutoides*, a single chromosome arm shows homology to the X chromosome of the house mouse, indicating the presence of an X-autosome translocation, whereas a whole acrocentric chromosome corresponds to the X of *Mus indutus*. The insert on (**b**) represents the (1.X) translocation of individual TK164752 *Mus minutoides*, with the long arm corresponding to the X chromosome.

Karyotypes for individuals in the *Mus minutoides* clade exhibited a diploid number of 34 and fundamental number (as defined by Veyrunes et al. 2004 as the total number of chromosomal arms per diploid genome, instead of number of autosomal arms) of FN=36 (Fig. 3a–d, Table 2). All autosomes were acrocentric in morphology, including the pair 13, which presented a small short arm in some metaphase spreads. The metacentric X chromosome is the largest element of the chromosome complement, followed by the subtelocentric Y chromosome, which is comparable in size with the first autosomal pair. Individuals in the *Mus indutus* clade exhibited diploid and fundamental numbers of 36 (Fig. 3e–f, Table 2). All chromosomes had an acrocentric morphology. Due to the lack of male karyotyped specimens, the Y chromosome morphology could not be determined. The FISH with *Mus* X whole chromosome probe allowed the detection of an X-autosome translocation on the karyotypes of *Mus minutoides* specimens (Fig. 3b, d), but not for individuals of *Mus indutus* (Fig. 3e). Banding results indicate that individuals of *Mus minutoides* from Botswana share the same sex-chromosome translocations (X.1) and (Y.1) as *Mus minutoides* from South Africa, although differential condensation of the South African chromosomes makes direct comparison difficult (Fig. 3a and b).

**Table 2.** Individuals of *Mus (Nannomys)* collected in Botswana including GenBank number, final species identification, gender determined in the field, museum preparation type (Alcoholic=alc; skin, skull, postcranial skeleton=SSPS; or Skull only), collection date, total length (TL), tail length (T), hind foot (HF), ear (E), weight in grams, karyotype, and sex-chromosome.

Genbank No.	Species	Gender “Field”	Prep. Type	Coll. Date	TL	T	HF	E	Weight (g)	Karyotype	Gender “Lab”
KF184315	*Mus indutus*	Female	SSPS	16-Jul-09	95	42	13	13	4,5	2n=36, FN=36	XX
KF184316	*Mus indutus*	Female	SSPS	22-Jul-09	85	40	10	10	2,9	none	-
KF184317	*Mus indutus*	Female	SSPS	27-Jul-09	101	43	14	11	5,1	none	-
KF184318	*Mus indutus*	Female	SSPS	22-Jul-09	14	45	12	10	6,3	2n=36, FN=36	XX
KF184319	*Mus indutus*	Female	SSPS	15-Jul-09	110	45	13	11	6,75	2n=36, FN=36	XX
KF184320	*Mus indutus*	Male	SSPS	18-Aug-11	98	45	15	10	4	none	-
KF184321	*Mus indutus*	Female	Alc	25-Aug-11	75	[23]	14	10	3	none	-
KF184322	*Mus indutus*	Female	Skull Only	17-Aug-11	109	40	15	11	5	none	-
KF184323	*Mus indutus*	Male	SSPS	20-Jul-09	86	42	14	12	4	none	-
KF184308	*Mus minutoides*	Female	SSPS	20-Jul-09	107	43	14	12	5,5	2n=34, FN=36	XX
KF184309	*Mus minutoides*	Male	SSPS	24-Jul-09	[80]	[23]	13	11	4,6	2n=34, FN=36	XY
KF184310	*Mus minutoides*	Female	SSPS	16-Aug-11	93	45	13	10	3,5	2n=34, FN=36	XY
KF184311	*Mus minutoides*	Male	SSPS	26-Jun-08	102	47	14	12	5,8	none	-
KF184312	*Mus minutoides*	Female	SSPS	15-Jul-09	111	52	15	11	5,5	2n=34, FN=36	XX
KF184313	*Mus minutoides*	Female	SSPS	20-Jul-09	99	44	12	9	4	2n=34, FN=36	XY
KF184314	*Mus minutoides*	Female	SSPS	26-Jul-09	96	47	14	10	3,7	2n=34, FN=36	XY

## Discussion

Efforts to resolve the geographic distributions of African pygmy mice remain in a state of flux. Regional studies involving DNA sequence data and karyotypes, such as presented here, contribute to a broader understanding of this complex genus. Historical (see Schmidt et al. 2008) and recent (Ferguson et al. 2010) bioinventories have resulted in extensive collections of *Mus* from Botswana, but there has been little consensus as to whether both *Mus minutoides* and *Mus indutus* occur in the country.

Mitochondrial sequence and cytogenetic data confirm the presence of both *Mus minutoides* and *Mus indutus* in Botswana. These specimens represent the first DNA sequences for these two species in Botswana, which we also made available for use in a recent paper by Lamb et al. (2014). Despite previous suggestions that *Mus minutoides* and *Mus indutus* occur in allopatry, our results confirm that these two species occur in sympatry and even syntopy in northwestern Botswana. Interestingly, we also found two lineages of *Mus minutoides* in northwestern Botswana (Koanaka Hills) that were 5% different in *cytb* sequence variation. We hypothesize that these two mitochondrial lineages were separated in the past and have now come back together in a region of secondary contact in the arid savannah region near the Kalahari Desert, a hypothesis that should be tested with broader sampling and using additional genetic markers.

Also of interest is the fact that no *Mus setzeri* were collected from either the Koanaka Hills or Gcwihaba Caves although their current range – as delimited by Monadjem and Coetzee (2008) and Skinner and Chimimba (2005) – includes this region of Botswana. We compared our specimens with *Mus setzeri* deposited at the National Museum of Natural History, Smithsonian Institution, Washington D.C., USA and found no evidence that any of our individuals correspond to this conspicuous form. Our failure to capture *Mus setzeri*,in spite of concerted trapping efforts in this region (> 2600 Sherman trap nights, > 280 pitfall trap nights during June 2008 and July 2009 seasons), is in agreement with Monadjem (2013b) who pointed to the scarcity of this species in collections as evidence for true ecological rarity. Further sampling is clearly warranted to more accurately delimit the exact geographic boundaries of *Nannomys* species both within Botswana and throughout the broader Southern African Subregion (Skinner and Chimimba 2005).

*Mus minutoides* in Botswana exhibit the 2n=34 karyotype with the diagnostic (X.1) and (Y.1) sex-autosome translocations that have also been documented in specimensfrom South Africa (Veyrunes et al. 2010), Zambia, Kenya (Castiglia et al. 2002, 2006), Central African Republic, and Ivory Coast (Jotterand-Bellomo 1984, 1986). Veyrunes et al. (2004) propose that 2n=34 with the 1 sex chromosome translocation is the ancestral karyotype for *Mus minutoides* and our results provide further support for an early (X.1) translocation before the radiation of *Mus minutoides* over a large geographic area. Furthermore, the 2n=34 cytotype is reported in several locations in northern South Africa, but not in southern South Africa or in other countries to the north, including Botswana. The fact that our sampling localities included individuals from the easternmost and northwestern regions of Botswana might be an indicator that this is the predominant cytotype in the country, likely extending into the bordering countries of Zambia, Zimbabwe, and Namibia.

We found that three of our gender identifications made in the field (Table 2, “Gender Field”) did not match the identifications made from karyotype assessments (Table 2, “Gender Lab”) indicating the potential for X*Y females. Therefore, we attempted to examine these specimens for the possibility of sex reversal in *Mus minutoides*, which has been documented in other countries (Veyrunes et al. 2013). Although we have tried to identify the X* chromosome in our samples through X chromosome morphology assessment as well as DAPI banding patterns, the particular high degree of condensation of the chromosomes in our *in vivo* bone marrow preparations did not allow us to ascertain the nature of the X chromosomes of two of these three specimens. For one of the individuals, both the morphology and banding patterns of the X chromosome do not seem to correspond to those of the derivative X* chromosome (Fig. 3a), indicating that field misidentification of sex might have been the case for that specimen (the reproductive organs can no longer be clearly seen on the prepared skin of this specimen). Additionally, there were no evident X chromosome polymorphisms in the XX female specimens, which would be expected in populations where X*Y females were present. Due to our small sample, and the relative low frequency of the X* found in populations outside South Africa, we were not able to rule out the presence of the X polymorphism in Botswana. Further collecting efforts, together with an in depth sex determination study, including high quality chromosome preparations suitable for G-banding studies, will be needed to shed further light on this issue.

Our data presented here agree with previous molecular phylogenies of *Nannomys*, with well-defined clades representing *Mus minutoides* and *Mus indutus* exhibiting diploid and fundamental numbers consistent with those reported in the literature. Veyrunes et al. (2010) detected a wide range of chromosomal variation for *Mus minutoides* in South Africa, with one particular clade presenting 2n=34, FN=36. Our *Mus minutoides* samples display chromosome conservation as well as sequence similarity to the South African clade bearing karyotypic stasis, indicating that these specimens might be part of a widespread group chromosomally and genetically isolated from the karyotypically diverse 2n=18 *Mus minutoides* clade. *Mus indutus* on the other hand exhibits a karyotype not very divergent from the proposed ancestral karyotype for *Nannomys* (2n=36 with all acrocentric chromosomes; Veyrunes et al. 2004), similar to many of the basal lineages included in recent molecular phylogenies (see Britton-Davidian et al. 2012).

## Appendix

**Table:** Individuals included in the molecular phylogeny representing eleven species with the country of origin, GenBank number and the original citation for the original description. RCA = Central African Republic.

*Mus (Nannomys)*	Country	Genbank No.	Reference
*Mus baoulei*	Benin	EU603991-92	Kan Kouassi et al. 2008
	Guinea	EU603995	Kan Kouassi et al. 2008
	Ivory Coast	EU603993-94, 98	Kan Kouassi et al. 2008
*Mus bufo*	Burundi	DQ789905	Mboumba et al. 2011
*Mus haussa*	Chad	AJ875071	Veyrunes et al. 2005
	Mali	AJ698877	Chevret et al. 2005
	Niger	AJ875072-73	Veyrunes et al. 2005
	Senegal	AJ875074	Veyrunes et al. 2005
*Mus indutus*	Botswana	KF184315-23	This paper
	South Africa	AJ698874	Chevret et al. 2005
	South Africa	AJ875070	Veyrunes et al. 2005
*Mus mattheyi*	Burkina Faso	AJ877114	Veyrunes et al. 2005
	Guinea	EU603970-73	Kan Kouassi et al. 2008
	Mali	AJ698876	Chevret et al. 2005
	Mali	AJ875066-67	Veyrunes et al. 2005
	Senegal	AB125781	Suzuki et al. 2004
	Senegal	AJ875068	Veyrunes et al. 2005
	Togo	AJ875069	Veyrunes et al. 2005
*Mus minutoides*	Botswana	KF184308-14	This paper
	Congo	DQ789929	Mboumba et al. 2011
	Gabon	DQ789911, 20, 26	Mboumba et al. 2011
	Guinea	AJ875076-77	Veyrunes et al. 2005
	Guinea	EU603936-37, 60-61, 64-65	Kan Kouassi et al. 2008
	Ivory Coast	EU603925-28, 30-33, 35, 45, 47, 49, 54-56, 58, 999, 001-02, 005	Kan Kouassi et al. 2008
	Kenya	AJ875084	Veyrunes et al. 2005
	Kenya	AY057816	Lundrigan et al. 2002
	RCA	DQ789938-39	Mboumba et al. 2011
	South Africa	AJ875078-80	Veyrunes et al. 2005
	South Africa	FN985222-24	Veyrunes et al. 2010
	Tanzania	AJ875081	Veyrunes et al. 2005
*Mus musculoides*	Cameroon	HM635855-56	Dobigny et al. 2011
	Guinea	EU603968-69	Kan Kouassi et al. 2008
	Guinea	DQ789902	Mboumba et al. 2011
	Ivory Coast	EU603967	Kan Kouassi et al. 2008
	Ivory Coast	DQ789901	Mboumba et al. 2011
	Mali	Z96069	Barome et al. 1998
	Mali	AJ698875	Chevret et al. 2005
	Mali	AJ875075	Veyrunes et al. 2005
	Mali	JX292892-93	Schwan et al. 2012
*Mus setulosus*	Cameroon	EU603989	Kan Kouassi et al. 2008
	Cameroon	DQ789900	Mboumba et al. 2011
	Gabon	AJ698873	Chevret et al. 2005
	Guinea	AJ875083	Veyrunes et al. 2005
	Guinea	EU603976, 78, 82-83, 86	Kan Kouassi et al. 2008
	Ivory Coast	EU603974-75, 77, 79-81, 84-85, 88, 97	Kan Kouassi et al. 2008
	Ivory Coast	GU830865, 67, 69	Coulibaly-N’golo et al. 2011
	RCA	AJ875082	Veyrunes et al. 2005
	RCA	EU603990	Kan Kouassi et al. 2008
*Mus sorella*	RCA	DQ789904	Mboumba et al. 2011
*Mus* sp.	Chad	AJ875085	Veyrunes et al. 2005
*Mus tenellus*	Ethiopia	DQ789903	Mboumba et al. 2011

## Tables 1, 2, and Appendix

These are the tables (1 and 2) and Appendix.
